# Serum Creatinine-to-Cystatin C Ratio in the Progression Monitoring of Non-alcoholic Fatty Liver Disease

**DOI:** 10.3389/fphys.2021.664100

**Published:** 2021-04-07

**Authors:** Shaobo Li, Jing Lu, Geng Gu, Wenkun Bai, Yafen Ye, Yuqian Bao, Haoyong Yu, Junfeng Han

**Affiliations:** ^1^Department of Endocrinology and Metabolism, Shanghai Jiao Tong University Affiliated Sixth People’s Hospital, Shanghai Clinical Center for Diabetes, Shanghai Diabetes Institute, Shanghai Key Laboratory of Diabetes Mellitus, Shanghai, China; ^2^Department of Radiology, Shanghai Jiao Tong University Affiliated Sixth People’s Hospital, Shanghai, China; ^3^Department of Ultrasound in Medicine, Shanghai Jiao Tong University Affiliated Sixth People’s Hospital, Shanghai Institute of Ultrasound in Medicine, Shanghai, China

**Keywords:** biomarker, disease progression, non-alcoholic fatty liver disease, psoas muscles, visceral obesity

## Abstract

**Background:**

The simultaneous assessment of visceral adiposity and muscle mass might be useful to monitor the risk of non-alcoholic fatty liver disease (NAFLD) progression in large population. We aimed to investigate the value of serum creatinine-to-cystatin C ratio (CCR) in evaluating these two parameters and predicting liver steatosis and fibrosis.

**Methods:**

154 overweight/obese inpatients (49 males, 105 females) scheduled for bariatric surgery and 49 non-overweight/obese volunteers (18 males, 31 females) responded to the hospital advertisement were involved in the cross-sectional study. Liver steatosis and fibrosis were diagnosed with transient elastography (TE). The psoas muscle area (PMA) and visceral fat area (VFA) were measured using magnetic resonance imaging.

**Results:**

The body mass index, insulin resistance, and lipid profiles showed significant differences between the CCR tertiles. Multiple regression analyses revealed that the CCR was significantly associated with the controlled attenuation parameter (β = −0.30, *P* = 0.006 in males; β = −0.19, *P* = 0.017 in females) and liver stiffness measurements in males (β = −0.246, *P* = 0.044). A low CCR was associated with moderate-to-severe steatosis (*P* < 0.001), significant liver fibrosis (*P* < 0.01), and excellent predictive power for these two conditions (*P* < 0.01). The CCR had a negative correlation with the VFA/PMA ratio (*r* = −0.584, *P* < 0.001 in males; *r* = −0.569, *P* < 0.001 in females).

**Conclusions:**

The CCR is a serum marker for muscle-adjusted visceral fat mass, and a low CCR is associated with an increased risk of progressive NAFLD.

## Introduction

Non-alcoholic fatty liver disease (NAFLD) is the most common chronic liver disease worldwide (Younossi et al., 2016). The global prevalence of NAFLD is reported to be 24–30% and increases together with that of obesity and diabetes mellitus (Fan et al., 2017). In 10–22% of patients, NAFLD progresses to non-alcoholic steatohepatitis, which is associated with high mortality from numerous liver and cardiovascular diseases (Bazick et al., 2015). To date, no pharmacotherapy exists for NAFLD. Therefore, it would be significant to identify high-risk NAFLD patients early and intensive monitoring of disease progression.

Obesity, particularly visceral obesity, is a major risk factor for the development and progression of NAFLD (Kim et al., 2016). However, about one in six NAFLD patients has a normal weight (Younossi et al., 2012). Therefore, the absolute amount of (visceral) fat mass might not adequately reflect the risk of NAFLD. Meanwhile, recent studies suggested that low muscle mass also contributes to the risk of NAFLD progression and severe liver fibrosis (Koo et al., 2017; Petta et al., 2017). Lower muscle mass can induce visceral fat accumulation and insulin resistance because of its key role in glucose homeostasis (Kim et al., 2018). Thus, simultaneous measurements of visceral fat and muscle mass might be an effective method for early screening and intensive monitoring of high-risk NAFLD populations.

Cross-sectional imaging at the level of the third lumbar vertebra (L3) was recommended by the National Institutes of Health to assess whole-body composition (Gomez-Perez et al., 2016). The psoas muscle area (PMA) has been shown to be an effective estimate of overall muscle mass and prognostic biomarker for poor health outcomes (e.g., all-cause mortality) (Mamane et al., 2016). However, the need for special equipment limits its clinical application. The serum creatinine-to-cystatin C ratio (CCR, also Sarcopenia Index) was first reported in 2013 and validated in subsequent studies as a biomarker of muscle mass (Tetsuka et al., 2013; Kashani et al., 2017; Barreto et al., 2019). Serum creatinine levels are proportional to muscle mass in patients with normal renal function (Barreto et al., 2019). Cystatin C is produced by all cells at a constant rate but shows elevated levels in obese individuals due to the enlarged visceral fat (Köttgen et al., 2008). Hence, the CCR might simultaneously reflect body visceral fat and muscle mass, but its ability to assess both these body components has not been established in studies. Furthermore, the relationship between the CCR and the course of disease in NAFLD is unknown to date. Therefore, we aimed to investigate a potential association of CCR with the degree of steatosis and fibrosis in NAFLD and body composition in a Chinese population.

## Materials and Methods

### Subjects

This cross-sectional study involved 49 non-overweight/obese volunteers (18 males and 31 females) and 154 overweight/obese inpatients (49 males and 105 females). Among the inpatients scheduled for an upcoming bariatric surgery at our institution between February 2019 and April 2020, 169 inpatients aged 18–67 years old were eligible for this study. After applying the following inclusion criteria: normal renal function (creatine ≤115 μmol/L, glomerular filtration rate >60 mL/min, without hemodialysis), 162 inpatients were included. Based on the exclusion criteria: (1) liver disease of different or mixed etiologies (excessive alcohol intake, hepatitis B, hepatitis C, autoimmune liver disease, etc.); (2) use of steatosis-inducing drugs or immunosuppressive drugs; (3) pregnancy, abnormal thyroid function, or malignant disease. We excluded eight patients. We also recruited 49 healthy volunteers with normal weight who responded to the hospital advertisements. Consequently, we enrolled 154 inpatients and 49 volunteers with or without NAFLD in the study.

### Clinical and Laboratory Assessments

All subjects completed a questionnaire about their demographics and health (such as age, medical history, and drinking of alcohol history). Height and body weight were measured with a digital scale. The waist circumference was measured at the midpoint between the lower rib margin and the iliac crest. The BMI was calculated as body weight (kg) divided by height squared (m^2^). Blood pressure was measured after 5 min of rest.

A 12-h overnight fasting blood sample was drawn from an antecubital vein to determine the liver enzymes, lipid profile, kidney function, fasting plasma glucose (FPG), hemoglobin A1c (HbA1c), and insulin serum concentrations in all subjects. Serum creatinine levels were assessed with the sarcosine oxidase method, and cystatin C was measured using an immunonephelometric assay. The CCR was then calculated as the serum creatine concentration (μmol/L) divided by the cystatin C concentration (mg/L). Based on the above data, we calculated insulin resistance (HOMA-IR) (Matthews et al., 1985) and determined the fatty liver index, Fibrosis-4 index, NAFLD fibrosis score, and BARD score as previously described (Vilar-Gomez and Chalasani, 2018).

### Psoas Muscle Area and Visceral Fat Measurement

All subjects were examined in the supine position with the Achieva 3.0T MRI system (Philips Healthcare, Eindhoven, The Netherlands). A medically trained technician selected one 10-mm slice at the L3 level with good contrast for the PMA and visceral fat area (VFA) measurements using the sliceOmatic 5.0 software (TomoVision, Magog, Canada). The software calculates areas of different tissues and expresses the measurements in cm^2^ based on the previously reported method (Shen et al., 2004).

### Controlled Attenuation Parameter and Liver Stiffness Measurement

The controlled attenuation parameter (CAP, in dB/m) and liver stiffness measurements (LSM, in kPa) were captured simultaneously by using the FibroScan^®^ (Echosens, Paris, France) equipped with M- or XL-probes to assess liver steatosis and stiffness. Details of this examination have been described in previous publications (Sasso et al., 2012; Koehler et al., 2016). Fasted subjects were lying supine with their right hand on their head and were examined by one well-trained sonographer who was blinded to the subjects’ underlying liver conditions. CAP was only calculated when the liver stiffness measurement was valid in order to ensure an accurate attenuation. A reliable LSM was defined using the following 3 criteria: (1) more than 10 valid shots; (2) a success rate (SR: the ratio of valid shots to the total number of shots) of at least 60%; and (3) a ratio of the interquartile range (IQR) of liver stiffness to median value (IQR/M) <30%.

### Definitions Used in This Study

Type 2 diabetes was defined as a fasting glucose level ≥7.0 mmol/L (126 mg/dL), HbA1c ≥6.5%, or taking antidiabetic medications (American Diabetes Association, 2018). Overweight (BMI ≥ 24.0 to BMI < 28.0) and obesity (BMI ≥ 28.0) were determined in accordance with the standard definitions proposed by the Working Group on Obesity in China (Zhou, 2002). NAFLD was defined as the presence of at least two of three abnormal findings on abdominal ultrasonography without evidence of any possible causes of liver diseases according to the diagnostic guidelines for NAFLD in the Asia-Pacific region: hepatorenal echo contrast, deep attenuation, and vascular blurring (Farrell et al., 2007). For this study, moderate-to-severe steatosis was defined as a CAP value greater than 268 dB/m (Tsai and Lee, 2018), significant liver fibrosis was defined as the presence of steatosis and LSM ≥8.0 kPa (Roulot et al., 2017; Alferink et al., 2019).

### Statistical Analyses

Statistical analysis was performed using IBM SPSS Statistics for Windows, version 25.0 (IBM Corp., Armonk, NY, United States) and GraphPad Prism 8 (GraphPad Software, San Diego, CA, United States). All data were tested for normality using the Shapiro-Wilk test. Categorical variables were presented as number (percentages). Continuous variables with normal and non-normal distributions were presented as mean ± standard deviation (SD) and medians (IQR), respectively. We performed subgroup analyzes for the CCR tertiles by sex as follows to avoid potential sex influences: for males, lowest tertile <75.56; middle tertile 75.56–100.00; highest tertile >100.00; for females, lowest tertile <68.89; middle tertile 68.89–98.00; highest tertile >98.00.

Differences between groups were assessed using the Chi-square or Fisher’s exact test for categorical variables and the One-way ANOVA analysis or Kruskal-Wallis Test for continuous factors. Parameters associated with CAP and LSM were identified by multiple stepwise linear regression analysis. Logistic regression models were used to analyze a potential association of CCR with moderate-to-severe steatosis (CAP > 268 dB/m) and significant liver fibrosis (LSM ≥ 8.0 kPa). The ability of CCR to identify these liver conditions was evaluated by receiver operating characteristic (ROC) analysis. Correlation coefficients were calculated using simple linear regression analysis to assess the associations of CCR with the TE and abdominal MRI measurements. A *P*-value < 0.05 was defined as statistically significant.

## Results

### Comparison of Anthropometric and Metabolic Characteristics Between Subjects in Different CCR Tertiles

Compared with the subjects in the lowest CCR tertile, those in the middle and highest CCR tertile had statistically significantly lower BMI, waist circumference, blood pressure, cystatin C, FPG, triglycerides (TG), low-density lipoprotein cholesterol, HOMA-IR, HbA1c, serum insulin levels and prevalence of lifestyle-related diseases (*P* < 0.001), including NAFLD, type 2 diabetes, and obesity (Table 1). Conversely, serum creatinine and high-density lipoprotein cholesterol (HDL-c) were significantly higher in the middle and highest CCR tertile compared to the lowest. Moreover, the VFA and VFA/PMA ratio were significantly higher in the lowest CCR tertile than the middle and highest tertile, while the PMA did not differ significantly among the three groups.

**TABLE 1:** The demographic and anthropometrics characteristics, insulin resistance and lipid profiles, hepatic conditions, markers of steatosis and fibrosis, and lifestyle-related diseases for 203 participants with tertile stratification according to the CCR.

	**1st Tertile**	**2nd Tertile**	**3rd Tertile**	***F*/χ^2^ value**	***P*-value**
N (M/F)	67 (22/45)	67 (22/45)	69 (23/46)		
Age (y)	33.3111.78	31.398.96	30.226.11	1.951	0.145
BMI (kg/m^2^)	37.396.61	34.947.72	27.358.11	33.116	< 0.001
WC (cm)	115.2514.56	108.3018.34	90.0819.91	36.517	< 0.001
SBP (mmHg)	131.5715.61	130.6814.21	118.9415.93	14.374	< 0.001
DBP (mmHg)	86.3011.11	87.1712.67	80.1013.20	6.549	0.002
Scr (μmol/L)	53.9111.77	61.9313.24	68.0013.69	20.274	< 0.001
CysC (mg/L)	0.870.16	0.730.15	0.580.11	71.449	< 0.001
CCR	62.127.17	84.578.72	118.0717.70	363.358	< 0.001
Male	66.246.45	87.717.67	126.0420.77	114.257	< 0.001
Female	60.116.68	83.048.88	114.0814.62	295.498	< 0.001
VFA	175.9167.07	145.3074.84	94.5482.41	18.980	< 0.001
PMA	27.067.82	25.988.17	25.079.11	0.887	0.414
VFA/PMA	6.802.60	5.612.78	3.592.65	23.184	< 0.001
**Insulin resistance and lipid profiles**					
FPG (mmol/L)	6.692.46	6.452.42	5.291.71	7.680	0.001
HbA1c (%)	7.021.97	6.782.06	5.761.11	9.812	< 0.001
HOMA-IR	8.12 (4.41,11.91)	6.19 (2.63, 10.22)	1.92 (1.36, 5.54)	42.121	< 0.001
FINS (μU/ml)	29.44 (16.61,42.16)	20.02 (12.16, 33.83)	10.06 (6.44, 21.88)	35.008	< 0.001
TC (mmol/L)	4.770.74	5.041.08	4.690.97	2.641	0.074
TG (mmol/L)	2.201.49	1.931.27	1.170.80	13.012	< 0.001
HDL-c (mmol/L)	1.030.27	1.160.33	1.470.48	25.256	< 0.001
LDL-c (mmol/L)	2.830.62	3.050.88	2.620.75	5.370	0.005
**Hepatic conditions**					
CAP (dB/m)	344.1246.21	323.3963.27	268.9669.82	27.914	< 0.001
LSM (kPa)	8.124.82	7.104.03	5.953.22	4.864	0.009
Albumin (g/L)	43.673.18	43.513.71	45.142.42	5.633	0.004
AST (U/L)	27.0 (17.0, 45.0)	26.0 (18.0, 42.0)	18.0 (16.0, 25.5)	11.813	0.001
ALT (U/L)	36.0 (18.0, 68.0)	43.0 (21.0, 84.0)	22.0 (15.0, 41.0)	14.725	0.001
γ-GT (U/L)	38.0 (25.0, 58.0)	35.0 (20.0,52.0)	16.0 (12.0, 35.5)	31.453	< 0.001
**Markers of steatosis and fibrosis**					
Platelet (10^9^/L)	273.2465.74	277.4969.01	273.9043.36	0.097	0.908
FLI	21.85 (5.67, 65.51)	16.60 (1.85, 57.60)	0.14 (0.03, 6.27)	51.932	< 0.001
FIB4-index	0.52 (0.38, 0.80)	0.46 (0.35, 0.65)	0.46 (0.35, 0.53)	4.959	0.084
NFS	1.07 (0.11, 2.10)	0.40 (-0.57, 1.49)	-0.87 (-1.26, -0.02)	44.254	< 0.001
BARD score	2.371.09	1.901.05	1.740.93	7.052	0.001
**Lifestyle-related diseases**					
NAFLD (%)	64 (95.5)	59 (88.1)	42 (60.9)	29.848	< 0.001
T2DM (%)	38 (56.7)	31 (46.3)	12 (17.4)	23.612	< 0.001
Obesity (%)	65 (97.0)	56 (83.6)	30 (43.5)	57.839	< 0.001

*BMI, body mass index; WC, waist circumference; SBP, systolic blood pressure; DBP, diastolic blood pressure; Scr, serum creatinine; CysC, cystatin C; VFA, visceral fat area; PMA, psoas muscle area; FPG, fasting plasma glucose; HbA1c, hemoglobin A1c; HOMA-IR, homeostasis model assessment of insulin resistance; FINS, fasting insulin level; TC, total cholesterol; TG, total triglycerides cholesterol; HDL-c, high-density lipoprotein; LDL-c, low-density lipoprotein; CAP, controlled attenuation parameter; LS, liver stiffness; ALT, alanine aminotransferase; AST, aspartate aminotransferase; γ-GT, gamma-glutamyl transferase; FLI, fatty liver index; NFS, NAFLD fibrosis score; FIB-4 index, fibrosis-4 index.*

### Liver Function, Steatosis, and Fibrosis in Subjects Within the Different CCR Tertiles

Compared to the subjects in the lowest CCR tertile, subjects in the middle and highest CCR tertile presented with lower CAP and LSM and lower aspartate transaminase, alanine aminotransferase, and γ-glutamyl transferase levels, meaning that the most severe liver steatosis, fibrosis, and/or inflammation was present in the lowest CCR tertile (Table 1). Further analysis revealed that the fatty liver index, NAFLD fibrosis score, Fibrosis-4 index, and BARD score were statistically significantly higher in subjects in the lowest CCR tertile than in the middle and highest tertile.

### NAFLD-Related Factors Associated With CAP and LSM in Males and Females

Multivariate linear regression identified HDL-c, HOMA-IR, CCR, and TC as independent determinants of CAP in males, whereas TG, HDL-c, alanine aminotransferase, and CCR were independently associated with CAP in females (Table 2). The CCR was an independent factor affecting LSM in males but not in females.

**TABLE 2:** Multiple stepwise linear regression analysis for NAFLD pathophysiological factors associated with liver steatosis and stiffness measured by elastography for 203 participants.

**Parameter**	**Multivariate analysis**			
	***B***	**Std. error**	**β**	***P*-value**
**Male group (*n* = 67)**				

**CAP measurement**				
HDL-c (mmol/L)	–68.36	17.64	–0.42	<0.001
HOMA-IR	2.88	0.87	0.32	0.002
CCR	–0.69	0.24	–0.30	0.006
TC (mmol/L)	14.44	5.17	0.28	0.007
**LS measurement**				
AST (U/L)	0.06	0.20	0.33	0.007
CCR	–0.04	0.02	–0.246	0.044

**Female group (*n* = 136)**				

**CAP measurement**				
TG (mmol/L)	19.04	4.69	0.31	<0.001
HDL-c (mmol/L)	–38.66	12.37	–0.24	0.002
ALT (U/L)	0.47	0.13	0.25	0.001
CCR	–0.53	0.22	–0.19	0.017
**LS measurement**				
AST (U/L)	0.07	0.01	0.45	<0.001
TG (mmol/L)	0.62	0.22	0.22	0.006

*β, standardized regression coefficient; CAP, controlled attenuation parameter; LS, liver stiffness; HDL-c, high-density lipoprotein; HOMA-IR, homeostasis model assessment of insulin resistance; CCR, calculated as serum creatinine divided by cystatin C; TC, total cholesterol; TG, total triglycerides cholesterol; AST, alanine aminotransferase; ALT, aspartate aminotransferase.*

### Risk of Liver Steatosis and Fibrosis of Subjects Within the Different Tertiles of CCR

Table 3 shows that if the risk of moderate-to-severe steatosis in the highest tertile was one, the relative risks were 8.346 (95% CI 3.654–19.066) in the middle tertile and 31.238 (95% CI 8.918–109.42) in the lowest tertile (unadjusted model). Moreover, the relative risks for significant liver fibrosis using the highest tertile as the reference were 3.481 (95% CI 1.416–8.560) in the middle tertile and 4.256 (95% CI 1.747–10.366) in the lowest tertile (unadjusted model). The results of the Model 1 and Model 2 analyses were similar, indicating that a CCR in the middle and lower tertile was an independent risk factor for liver steatosis and fibrosis in our subjects.

**TABLE 3:** Unadjusted and adjusted odds ratios with 95% confidence intervals of having moderate-to-severe steatosis and extensive liver stiffness by tertiles of CCR.

	**Unadjusted**		**Model 1**		**Model 2**	
	**OR (95%CI)**	***P***	**OR (95%CI)**	***P***	**OR (95%CI)**	***P***
**Moderate-to-severe steatosis (CAP > 268 dB/m)**						

CCR	0.947 (0.930–0.963)	<0.001	0.950 (0.933–0.967)	<0.001	0.964 (0.945–0.983)	<0.001
**Tertiles of CCR**						
3rd Tertile	1.0		1.0		1.0	
2nd Tertile	8.346 (3.654–19.066)	<0.001	10.046 (4.157–24.278)	<0.001	4.508 (1.555–13.072)	0.006
1st Tertile	31.238 (8.918–109.420)	<0.001	32.521 (9.000–117.506)	<0.001	7.756 (1.967–30.581)	0.003
*P* for trend test	<0.001		<0.001		<0.001	

**Significant liver fibrosis (LSM ≥ 8.0 kPa)**						

CCR	0.977 (0.963–0.991)	0.002	0.981 (0.966–0.995)	0.010	0.977 (0.961–0.992)	0.003
**Tertiles of CCR**						
3rd Tertile	1.0		1.0		1.0	
2nd Tertile	3.481 (1.416–8.560)	0.007	3.014 (1.199–7.580)	0.019	3.149 (1.231–8.055)	0.017
1st Tertile	4.256 (1.747–10.366)	0.001	3.503 (1.404–8.741)	0.007	3.686 (1.444–9.408)	0.006
*P* for trend test	0.002		<0.001		<0.001	

*All 203 participants (67 males and 136 females) were divided into the CCR tertiles according to gender. Model 1 adjusted for age, gender, FBG, HbA1C, and HOMA-IR. Model 2 adjusted for age, gender, FBG, HbA1C, HOMA-IR, TC, TG, HDL-C, and LDL-C.*

### CCR Cut-Off Value to Identify Liver Steatosis and Fibrosis

**FIGURE 1:**
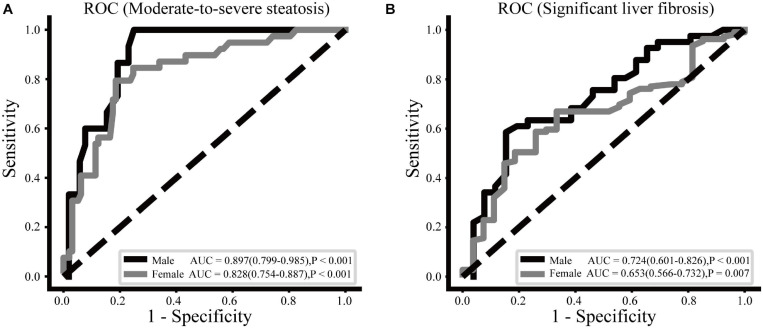
ROC curves of the CCR to moderate-to-severe steatosis **(A)** and significant liver fibrosis **(B)** for males and females.

The ROC curve analysis of CCR cut-off values indicating moderate-to-severe steatosis and significant liver fibrosis in males and females is shown in Figure 1. For moderate-to-severe steatosis, the cut-off value with the highest Youden index was a CCR of 94.44, with a sensitivity of 0.750 and a specificity of 1.000 in males, whereas the CCR cut-off value was 95.71 (sensitivity 0.814, specificity 0.795) in females. The CCR had acceptable power in males (AUC = 0.724, *P* < 0.001) and females (AUC = 0.653, *P* = 0.007) to identify significant liver fibrosis, with a cut-off value of 94.44 in males (sensitivity 0.846, specificity 0.585) and 73.33 in females (sensitivity 0.667, specificity 0.670).

### Correlation of CCR With the Measurements of TE and Abdominal MRI

**FIGURE 2:**
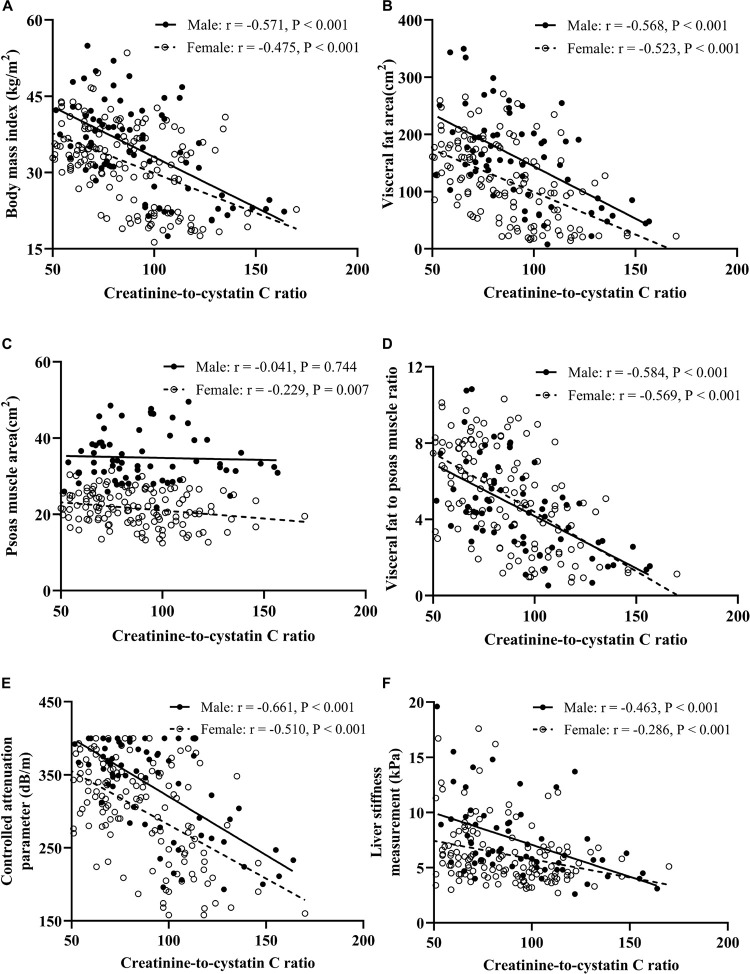
Correlation analysis between CCR and BMI **(A)**, visceral fat area **(B)**, psoas muscle area **(C)**, visceral fat to psoas muscle ratio **(D)**, controlled attenuation parameter **(E)**, and liver stiffness measurement **(F)**.

As shown in Figure 2, VFA/PMA ratio exhibited the largest correlation coefficient with the CCR than BMI and VFA in both males and females. The PMA was significantly associated with the CCR in females (*r* = −0.229, *P* = 0.007) but not in males. If males and females had a similar CCR, males tended to have a higher BMI, more visceral fat, and more muscle mass, but the VFA/PMA ratio between male and female was almost equal, which is manifested by the almost overlap of the linear regression cures. The CAP and LSM were significantly negatively correlated with the CCR, regardless of sex. In males and females with a similar CCR, males tended to have a higher CAP and LSM, indicating a sex difference in the association of the CCR with NAFLD.

## Discussion

This study was the first to investigate the relation between the CCR and liver steatosis and fibrosis. We found that a low CCR is significantly associated with high risk of moderate-to-severe steatosis and significant liver fibrosis both in males and females. This association remained significant even after adjustment for potential confounding factors. Moreover, our results indicate that the CCR is more suitable as a marker of muscle-adjusted visceral fat mass than measuring muscle mass, which supports the utility of the CCR in the monitoring of NAFLD.

Currently, the CCR is proposed as a marker of body muscle mass and sarcopenia. The association of the CCR with muscle mass and clinical outcomes (e.g., malnutrition, frailty, and hospital length of stay) has been reported in recent studies (Kashani et al., 2017; Barreto et al., 2019; Tabara et al., 2020). However, our study provides a new insight in that CCR is superior to muscle mass as a marker of muscle-adjusted visceral fat mass. In our study, subjects within the lowest CCR tertile exhibited a remarkable range of adiposity-associated characteristics, such as a high BMI, large waist circumference, and disorders of glucose and lipid metabolism. Importantly, the CCR had a highly negative correlation with the VFA/PMA ratio both in males and females, but the PMA did not show such relationship. The almost identical linear regression curves for the VFA/PMA ratio and CCR in both males and females further support the possibility of using the CCR as a biomarker for muscle-adjusted visceral fat mass. Previous studies have mainly focused on non-obese patients with small BMI ranges, which means the influence of skeletal muscle mass was potentially larger than that of visceral fat. Our subjects had a wide BMI range, which further highlights the significant role of visceral fat in the CCR. Moreover, changes in the number of visceral fat cells are the main source of changes in the number of whole-body cells in the adult, which are closely related to visceral fat accumulation (Spalding et al., 2008; Arner et al., 2010). Inflammation and oxidative stress, mainly induced by visceral fat accumulation, can increase the expression and secretion of cystatin C (Neeland et al., 2013; Xu et al., 2015). Overall, we hypothesize that visceral fat mass is the determinant of CCR, whereas muscle mass is more of an important correcting factor due to its inherent antagonistic function to visceral fat.

A recent cross-sectional study (Shida et al., 2018) reported that decreased skeletal muscle mass to VFA ratio was closely associated with an increased risk of moderate to severe steatosis and advanced fibrosis. The longitudinal study conducted by Mizuno et al. (2019) further demonstrated that an increase in the ratio of skeletal muscle mass to body fat mass was correlated with a reduction in liver fat accumulation and liver damage. In our study, the subjects within the lowest CCR tertile presented the highest risk of moderate-to-severe liver steatosis and significant liver fibrosis. In addition, CCR was negatively correlated with CAP and LSM in both males and females, which further underlines the ability of CCR to predict steatosis and fibrosis. There are multiple interactions between muscle and visceral fat in the onset and progression of NAFLD. Chronic inflammation, oxidative stress, and excessive free fatty acid level in the blood caused by visceral fat accumulation have various adverse effects on the liver and muscle metabolism, including impaired insulin sensitivity, dyslipidemia, and liver cell apoptosis (Stinkens et al., 2015; Kalinkovich and Livshits, 2017). Sufficient muscle mass can alleviate hepatic lipid accumulation by reducing free fatty acid levels in the blood and plays a key role in regulating glucose homeostasis in the body (Gemmink et al., 2020). Moreover, changes in myokine concentrations (e.g., irisin, interleukin-15, and brain-derived neurotrophic factor) may also play a critical role in NAFLD (Pedersen and Febbraio, 2012). It is worth noting that metabolically obese normal-weight populations and the elderly have a significant increase in NAFLD prevalence and liver fibrosis risk (Younossi et al., 2012, 2016). Exploring the associations between CCR and NAFLD by combining visceral fat and muscle mass measurements in these two groups may further our understanding of the pathophysiological mechanisms and assist in the timely identification of high-risk groups.

One strength of this study is that the CCR can be obtained by blood sampling and evaluates muscle-adjusted visceral fat mass at a lower cost than MRI, computed tomography, and other methods, which is a significant advantage for NAFLD risk monitoring in large populations. However, there were several limitations to this study. First, the cross-sectional design of the study made it impossible to identify causal or temporal relationships between the CCR and NAFLD. Further prospective research is warranted to confirm a potential causal relationship. Second, we did not consider the determination of exercise, dietary intake of meat, or medications, such as cimetidine. These factors may change creatinine levels and possibly influence the CCR. Finally, the gold standard for the diagnosis of NAFLD is liver biopsy rather than ultrasonography. However, a liver biopsy is invasive and may have complications. Moreover, ultrasound is a widely used screening tool with high sensitivity and specificity for moderate and severe steatosis (Dasarathy et al., 2009).

## Conclusion

In conclusion, the CCR is strongly associated with moderate-to-severe steatosis and significant liver fibrosis, indicating its usefulness as a marker for muscle-adjusted visceral fat mass. Considering the increasing prevalence of NAFLD worldwide and the lack of effective therapy, the CCR might be a promising and easy-to-measure serum marker in the screening and monitoring of the progression of NAFLD in public health settings. Large prospective studies are necessary to confirm whether CCR predicts NAFLD progression and to explore the role of the CCR in metabolically obese but normal-weight populations and the elderly.

## Data Availability Statement

The raw data supporting the conclusions of this article will be made available by the authors, without undue reservation.

## Ethics Statement

The studies involving human participants were reviewed and approved by The Ethics Committee of Shanghai Jiao Tong University Affiliated Sixth People’s Hospital. The patients/participants provided their written informed consent to participate in this study.

## Author Contributions

SL, HY, and JH conceived the project. SL analyzed the data, prepared the figures, and drafted the manuscript. YY and SL recruited participants and collected the data. JL and GG were responsible for abdominal magnetic resonance examination. WB assisted in transient elastography. YB, HY, and JH contributed to the discussion and revised the manuscript. All authors critically reviewed, edited, and approved the final manuscript.

## Conflict of Interest

The authors declare that the research was conducted in the absence of any commercial or financial relationships that could be construed as a potential conflict of interest.

## Abbreviations

CCR: creatinine-to-cystatin C ratio
NAFLD: non-alcoholic fatty liver disease
BMI: body mass index
PMA: psoas muscle area
VFA: visceral fat area
CAP: controlled attenuation parameter
LSM: liver stiffness measurement
TE: transient elastography
MRI: magnetic resonance imaging.
